# OPTC-5. Molecular signatures of podoplanin expressing glioblastoma cell subsets with putative role in cancer associated thrombosis and microthrombosis

**DOI:** 10.1093/noajnl/vdab070.026

**Published:** 2021-07-05

**Authors:** Nadim Tawil, Rayhaan Bassawon, Brian Meehan, Laura Montermini, Dongsic Choi, Ali Nehme, Hamed Najafabadi, Yasser Riazalhosseini, Nicolas De Jay, Claudia Kleinman, Tenzin Gayden, Cristiana Spinelli, Lata Adnani, Charles Couturier, Kevin Petrecca, Nada Jabado, Janusz Rak

**Affiliations:** 1 McGill University, Montreal, QC, Canada; 2 Research Institute of McGill University Health Center, Montreal, QC, Canada; 3 McGill University and Genome Quebec Innovation Centre, Montreal, QC, Canada; 4 Lady Davis Research Institute - Jewish General Hospital, Montreal, QC, Canada; 5 Montreal Neurological Institute and Hospital, Montreal, QC, Canada

## Abstract

Vascular anomalies, including thrombosis, are a hallmark of glioblastoma (GBM) and an aftermath of dysregulated cancer cell genome and epigenome. Upregulation of podoplanin (PDPN) by cancer cells has recently been linked to an increased risk of venous thromboembolism in glioblastoma patients. Thus, regulation of this platelet activating transmembrane protein by transforming events and release from cancer cells into the circulation are of considerable interest. We took advantage of single-cell and bulk GBM transcriptome dataset mining and investigated the pattern of PDPN expression across several databases. Our analysis indicated that PDPN is expressed by distinct (mesenchymal) glioblastoma cell subpopulations and is downregulated by oncogenic mutations of EGFR and IDH1 genes, via changes in chromatin modifications (EZH2) and DNA methylation, respectively. Additionally, we utilized isogenic and stem GBM cell lines, xenograft models in mice, ELISA assays for PDPN, tissue factor (TF), platelet factor 4 (PF4) and clotting activation markers (D-dimer), and multicolor nano-flow cytometry to show that GBM cells exteriorize PDPN and/or TF as cargo of exosome-like coagulant extracellular vesicles EVs. We also documented an increase of platelet activation (PF4) or coagulation markers (D-dimer) in mice harboring the corresponding PDPN- or TF-expressing glioma xenografts, respectively. While PDPN was a dominant regulator of systemic platelet activation, co-expression of PDPN and TF impacted local microthrombosis. Our work suggests that distinct cellular subsets drive multiple facets of GBM-associated thrombosis and may represent targets for diagnosis and intervention.

